# Impact of Exercise and Metabolic Disorders on Heat Shock Proteins and Vascular Inflammation

**DOI:** 10.1155/2012/836519

**Published:** 2012-12-17

**Authors:** Earl G. Noble, Garry X. Shen

**Affiliations:** ^1^School of Kinesiology, University of Western Ontario, London, ON, Canada; ^2^Diabetes Research Group, Department of Internal Medicine and Physiology, University of Manitoba, 835-715 McDermot Avenue, Winnipeg, MB, Canada R3E 3P4

## Abstract

Heat shock proteins (Hsp) play critical roles in the body's self-defense under a variety of stresses, including heat shock, oxidative stress, radiation, and wounds, through the regulation of folding and functions of relevant cellular proteins. Exercise increases the levels of Hsp through elevated temperature, hormones, calcium fluxes, reactive oxygen species (ROS), or mechanical deformation of tissues. Isotonic contractions and endurance- type activities tend to increase Hsp60 and Hsp70. Eccentric muscle contractions lead to phosphorylation and translocation of Hsp25/27. Exercise-induced transient increases of Hsp inhibit the generation of inflammatory mediators and vascular inflammation. Metabolic disorders (hyperglycemia and dyslipidemia) are associated with type 1 diabetes (an autoimmune disease), type 2 diabetes (the common type of diabetes usually associated with obesity), and atherosclerotic cardiovascular disease. Metabolic disorders activate HSF/Hsp pathway, which was associated with oxidative stress, increased generation of inflammatory mediators, vascular inflammation, and cell injury. Knock down of heat shock factor-1 (HSF1) reduced the activation of key inflammatory mediators in vascular cells. Accumulating lines of evidence suggest that the activation of HSF/Hsp induced by exercise or metabolic disorders may play a dual role in inflammation. The benefits of exercise on inflammation and metabolism depend on the type, intensity, and duration of physical activity.

## 1. Introduction

The stress response is a self-protective mechanism against environmental stresses which is mediated via a group of evolutionally conserved proteins, heat shock proteins (Hsp). Hsp regulate the conformation and functions of a large number of cellular proteins in order to protect the body from stress [1]. The expression of Hsp is mainly modulated by a common transcription factor, heat shock factor-1 (HSF1). The activity, translocation, and expression of HSF1 respond to environmental stresses, such as heat shock, wounds, oxidative stress, and radiation [2]. Exercise is associated with transient elevations of Hsp expression, body temperature, hormones, and oxidative stress, which may reduce inflammatory mediators [3]. Metabolic disorders in common chronic diseases (diabetes, metabolic syndrome, and atherosclerotic cardiovascular disease) are associated with a prolonged stress response as a consequence of oxidative stress, altered hormone levels, vascular inflammation, and cell injury [4]. Type 1 diabetes is a common autoimmune disease characterized by pancreatic *β*-cell destruction and insulin deficiency which can lead to poor circulation and vascular disease [5]. This paper summarized up-to-date knowledge on the relationship between stress responses, oxidative stress, and vascular inflammation under exercise or metabolic disorders. Selected literature searched using PubMed over a period from 1981 to 2012 is provided.

## 2. Heat Shock Proteins

Hsp has evolved to perform multiple roles within cells, organs, and organisms [1]. These ubiquitous proteins, which are found both inside and outside the cell [7], have a generalized function of interacting with other proteins, hence their designation as molecular chaperones [8]. These interactions may influence the structure of the client protein(s) so that it may be maintained in a conformation appropriate for functional folding, targeted for degradation, or altered as part of a signaling pathway. Most Hsp have a multitude of activities based upon their cellular location (including extracellular), the client proteins they interact with [9], and their phosphorylation status which may modulate their aggregation [10], their localization [11], or their activation of enzymatic pathways [12]. As a consequence, Hsp not only protect cells and organisms against proteotoxic stresses, but these proteins are also critical in normal functioning of several cellular processes [13]. Amongst those signaling pathways which involve Hsp are several which are implicated in regulation of immune and inflammatory systems [14, 15]. Although there is some controversy regarding their exact role [16], Hsp may activate the immune response [17] but also dampen the inflammatory pathways [14].

Hsp have normally been classified according to their molecular mass with small Hsp, such as *α*A- and *α*B-crystallin, Hsp20, 22, 25/27, and other Hsp60, the Hsp70 and 90 families and Hsp110, and their cochaperones (Table 1), often working in concert to maintain cell structure and function [6]. A new nomenclature has more recently been introduced for Hsp [18]; however, for the purposes of this paper, we will refer to the more common mass-based nomenclature (see Table 1).

## 3. Regulation of the Transcription of Hsp

The regulation of the transcription of Hsp is mainly through heat shock factors (HSF). HSF represents a family of transcription factors induced by both stressful and nonstressful stimuli. The fundamental structure of HSF has been well conserved from yeast to humans [2, 19]. Four isoforms of HSF have been reported. HSF1, 2, and 4 are present in humans. HSF1 is ubiquitously expressed in mammalian tissues and relatively abundant in heart, ovary, brain, and placenta [20]. HSF2 is expressed in very low levels in postnatal tissue [21], and HSF4 is mainly expressed in brain and lung [22]. Under basal conditions, HSF1 exists as a monomer. Under stress, HSF1 is converted to a trimer which is required for the binding to the responsive element (heat shock element) of HSF1 in the Hsp promoter. Phosphorylation of specific HSF1 residues is also required for activation [23, 24], and the multiple pathways potentially involved in these phosphorylations [25–28] probably provide tissue and stress specificity. A variety of stresses beside heat shock may activate or upregulate HSF1 [19, 29, 30]. Indeed, activation of HSF1 was detected in diet-induced atherosclerotic lesions in rabbits and humans [31, 32]. 

**Figure 1:**
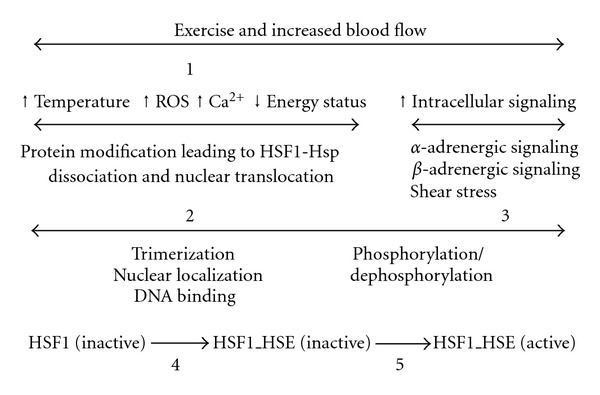
Schematic representation of activation of HSF1 with exercise and accompanying increases in vascular stress. Exercise initiates a number of factors, including elevations in temperature, reactive oxygen species (ROS), intracellular calcium (Ca^2+^), and decreased energy status [1], which may result in intracellular protein modification leading to dissociation of the heat shock transcription factor (HSF1) and heat shock proteins Hsp in the cytoplasm [2]. In addition, exercise activates adrenergic and shear stress intracellular signaling pathways [3]. Consequently HSF1 trimerizes and binds to heat shock elements (HSE) of nuclear DNA [4], whereupon specific phosphorylation/dephosphorylation events lead to a heat shock response [5]. Adapted from Noble, Melling, and Milne [6].

Given the importance of the heat shock response, it is not surprising that there are multiple redundant pathways by which the response may be activated [33]. Following exercise, it is likely that these pathways converge with the HSF1 through the translocation of the transcription factor from cytoplasm to nucleus [34, 35]. With exercise, likely candidates are the adrenergic stimuli associated with exercise operating through *α*- and *β*-adrenergic receptors [36–38] as well as elevated temperature and its attendant changes [39, 40] (see Figure 1).

## 4. Exercise and Hsp

Locke et al. [3] were the first to demonstrate that vigorous physical activity is associated with the induction of Hsp70 in rodents. Subsequently, increased expression of Hsp in humans following exercise was confirmed [41, 42]. As noted above, exercise is associated with many stressors, including elevated temperature, metabolic disturbances, altered calcium fluxes, increased production of reactive oxygen species (ROS), changed hormonal environment, and mechanical activation or deformation of tissues [13]. Exercise has also been described as inducing a mild inflammatory state [43]. The magnitude of the exercise stress, including whether it is acute or chronic, plays a major role in inducing the stress response. Generally, the more vigorous the exercise was, the greater the response was [40, 44–46]. Further, isotonic nondamaging contractions, such as these associated with endurance type activities, tend to lead to increases in Hsp60 and 70 with more limited responses in the small Hsp [3, 47]. In contrast, eccentric (often damaging) muscle contractions, also lead to increases, phosphorylation, and translocation of Hsp 25/27 and *α*B-crystallin [10, 48, 49]. These exercise-induced changes may be associated with protection of the mitochondria [50, 51], the sarcoplasmic reticulum [52], cytoskeletal protection [49], maintenance of enzymatic activity [53], and insulin sensitivity and glucose transport [54, 55]. With repetitive exercise (exercise training), an exercise-induced increase of Hsp70 is maintained whereas the initial response of other Hsp to exercise is diminished as training progresses [39].

Exercise involves the activation of specific muscles for movement but also requires the support of the neural, cardiovascular, and respiratory systems. The primary focus of investigators to date has been on skeletal and cardiac muscles. Such studies have suggested that in the sedentary state Hsp are expressed in a tissue-specific fashion [56]. Exercise is associated with changes in Hsp expression which are also specific to the Hsp in question [47, 49]. For example, Hsp70 almost always increases with exercise, whereas the cognate Hsc70 is not normally altered [57–60]. In a similar fashion, some tissues, such as myocardium, may demonstrate a more general response whereas skeletal muscle responds with fiber-specific changes [61, 62]. It is likely that differences in temperature reached during exercise [40] and the specific patters of muscle fiber activation [45] are responsible for some of these tissue-specific observations.

## 5. Exercise and Vascular Inflammation

Physical activity, or exercise, is known to improve overall health and protect against, delay the progress of, or ameliorate many common chronic diseases [63, 64], in particular those associated with whole body inflammation, including cardiovascular disease [65]. Although those individuals with the greatest cardiorespiratory fitness appear to benefit most [66], simply engaging in regular physical activity seems to be protective [67]. One of the primary targets that may benefit from increased physical activity is the vasculature [68–71]. Amongst the benefits of exercise on the vasculature are increased vasodilation and improved vascular compliance [72] which are likely a result of shear stress and cell stretch on both the endothelium and underlying smooth muscle [73, 74]. Exercise may protect the vasculature through a number of mechanisms [63, 68, 75] including reduced inflammation [76–79]. Short-term exercise reduces the levels of TNF-*α*, IL-6, plasminogen activator inhibitor-1 (PAI-1) [80], and cell adhesion molecules [81], protects against media-intimal hyperplasia [82, 83] and smooth muscle cell hypertrophy [83], and strengthens the endothelial barrier [84]. The anti-inflammatory role of exercise [43, 65, 78] is complicated; however, as intense unaccustomed exercise may be associated with increased cortisol [85], C-reactive protein [86], and modest increases in other proinflammatory cytokines [87].

Interestingly, heat shock exhibits beneficial effects on the vasculature which are similar to exercise, with reduced inflammation [88], reduced endothelial interaction with leukocytes [89], enhanced smooth muscle cell survival [90], and inhibition of myointimal hyperplasia and smooth muscle cell hypertrophy [91–95]. Although both heat shock and exercise are complex stressors likely leading to many changes in the integrated physiology of an organism, they both have some common characteristics including activation of stress hormones, ROS, and elevated temperatures leading to the activation of the heat shock response in a variety of tissues including the vasculature. Exercise increases ROS production, and ROS may play a signaling role to initiate the stress response [96]. Also, there is evidence that elevated temperature is critical for the activation of the heat shock response in exercising mammals [39, 40, 97, 98]. These similarities suggest that the protection conferred by exercise against myocardial ischemia-reperfusion injury [99] could be partially a consequence of the vascular expression of Hsp [100, 101]. Indeed, exercise leads to a rapid transcription of Hsp70 mRNA in the vasculature of rodents [62] which eventually results in protein accumulation [102, 103].

## 6. Vascular Function of Hsp

**Figure 2:**
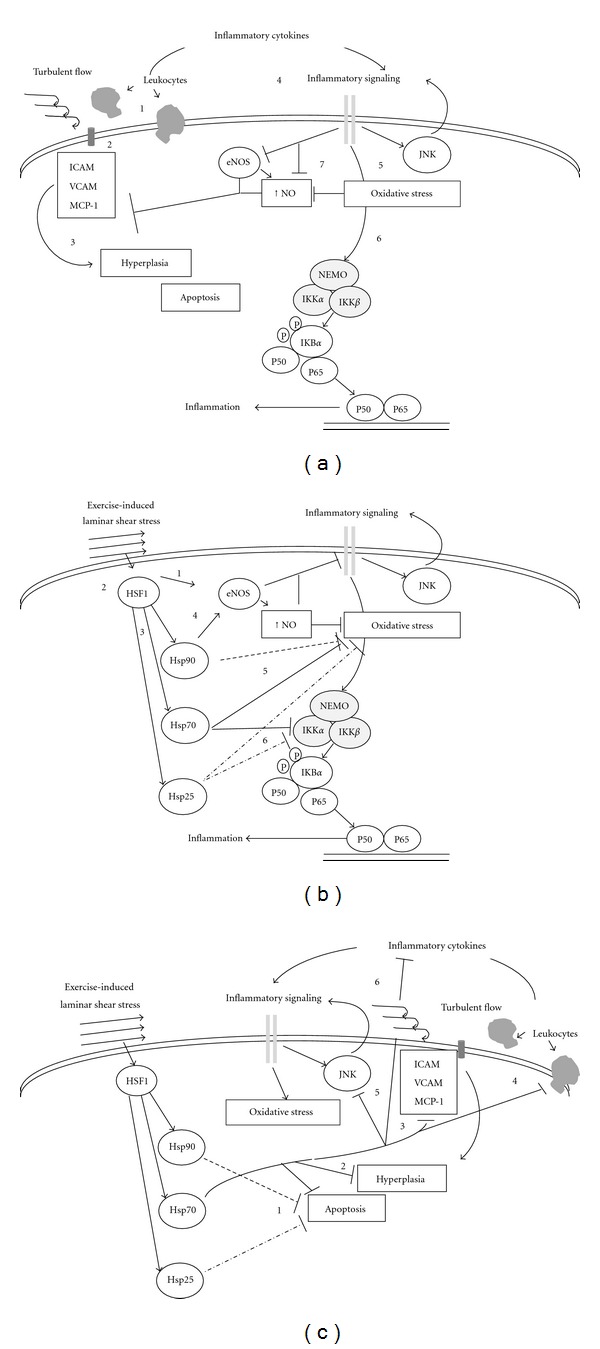
Scheme for relationships between exercise-associated hemodynamic changes, inflammatory response, and Hsp. (a) Low or turbulent flow is associated with leukocyte extravasation [1] and expression of adhesion molecules [2], resulting in intimal hyperplasia, cell apoptosis [3] and inflammatory signaling [4]. The associated inflammatory signaling leads to increased oxidative stress, induction of inflammatory pathways such as c-Jun NH_2_-terminal kinase (JNK) [5] and NF-*κ*B [6], and suppression of endothelial nitric oxide (eNOS) and oxidation of nitric oxide (NO) [7]. (b) In contrast, an exercise induced increase in laminar shear stress activates eNOS [1] and HSF1 [2]. HSF1 activation leads to increased heat shock proteins 25, 70, and 90 (Hsp25, Hsp70, and Hsp90) [3] which may inhibit many of these inflammatory processes indirectly via activation of eNOS signaling (Hsp90) [4] and directly through suppression of oxidative stress (Hsps 25, 70, and 90) [5] and inflammatory signaling including via the NF-*κ*B pathway (Hsps 25 and 70) [6]. (c) Hsp may also directly reduce apoptosis (Hsps 70 and 90) [1] and hyperplasia (Hsp 70) [2]. Hsp70 has further been implicated in decreased expression of adhesion molecules [3] leading to a reduction of leukocyte extravasation [4] and expression of inflammatory cytokines [6]. Hsp70 also suppresses JNK signaling [5] further inhibiting inflammatory signaling and cytokine release. See text for a more complete description. →— represents activating role; *|*— represents inhibitory role; - - - -: Hsp90 effects; ——: Hsp70 effects; -·-·-·-·: Hsp25 effects.

As throughout the rest of the body, Hsp likely play specific roles within the vasculature. The response of the vasculature to shear stress is complicated. Laminar flow, such as that associated with exercise, induces positive vascular remodeling, whereas turbulent or low flow, such as that associated with vascular inflammation and atherosclerosis, leads to adhesion of blood borne molecules and inflammation [104] (see Figure 2). The increased laminar flow associated with exercise causes endothelial cell remodeling which includes the activation of a number of signaling pathways and either activation or enhanced expression of Hsp [73, 105–107]. Hsp25/27, which is phosphorylated in association with shear stress [105], is involved in cytoskeletal organization [108]. Hsp20 is associated with *α*B-crystallin in cardiac tissue [109], and both are involved in flow-mediated smooth muscle relaxation [110–112]. Indeed, Hsp25/27 and Hsp20/*α*B-crystallin may reciprocally assist in controlling venous tone [113]. Hsp70 may modulate vascular contractility through thick filament regulation [114], and Hsp90 is intricately involved in activation of endothelial nitric oxide synthase (eNOS) and the subsequent release of nitric oxide (NO) and vascular relaxation [115].

## 7. Activation of the Vasculature

Normal endothelium provides an effective barrier to foreign materials and does not interact with circulating factors. With a variety of chronic diseases, including atherosclerosis, metabolic syndrome, and diabetes, there is a subtle change in the endothelium which leads to their “activation” (see Figure 2(a)). Initially, increased membrane permeability leads to the accumulation and modification of proteins, lipids, and lipoproteins on endothelium [116]. The endothelium then becomes “sticky,” exhibiting proinflammatory markers such as monocyte chemotactic protein-1 (MCP-1), vascular and intracellular cell adhesion molecules (VCAM-1 and ICAM-1, resp.,) and greater nitrotyrosine content [117, 118]. This leads to the recruitment of blood borne cells which infiltrate the intima resulting in macrophages evolving to foam cells leading to further inflammation and release of pro-coagulant factors, smooth muscle cell death and migration, and the eventual formation of an atherosclerotic plaque [116, 119]. During the course of this progressive dysfunction, NO availability plays a key role, as it is responsible for limiting many of the above processes. However, elevated oxidative stress associated with vascular inflammation leads to diminished NO availability [120]. Oxidation of the eNOS cofactor, tetrahydrobiopterin, uncouples eNOS such that superoxide rather than NO is formed [121]. This leads to NO scavenging to peroxynitrites and ultimately reduced activation of eNOS and an overall reduction in eNOS content [120, 122].

Although there are a variety of pathways by which inflammation can influence this vascular dysfunction, the nuclear factor kappa light chain enhancer of activated B cells (NF-*κ*B) pathway plays a critical role in this process [123–125] (see Figure 2(a)). NF-*κ*B has both anti- and proinflammatory roles; however, with progression of vascular damage, it primarily activates inflammatory pathways [123, 124, 126]. Members of the NF-*κ*B family, including p50, p52, p65, relB, and C-Rel, form homo- or heterodimers which are found in the cytoplasm in an inactive state bound to the inhibitor I*κ*B. Various stressors can release the I*κ*B from NF-*κ*B through a pathway which involves phosphorylation of I*κ*B by the IKK complex (IKK*α*, IKK*β*, and IKK*γ*). The phosphorylation of I*κ*B leads to its degradation by the ubiquitin proteasome pathway. The degradation of I*κ*B allows translocation of the NF-*κ*B dimer to the nucleus, where depending on the NF-*κ*B composition, recruited cofactors, and the sequence of targeted genes, variable responses may be observed [124, 127]. NF-*κ*B may also be activated via an IKK*α*-specific, noncanonical pathway [127]. Knockdown of HSF1 reduced Hsp27 expression and increased angiotensin II-induced NF-*κ*B activation in vascular smooth muscle cells [88]. This suggests that HSF1/Hsp 27 may mediate stress-activated vascular inflammation.

## 8. Anti-Inflammatory Actions of Hsp

It should be noted that Hsp, particularly extracellular Hsp, may play a key role in activating and exacerbating inflammation including vascular inflammation [17, 128–131] (see the following); however, given the anti-inflammatory phenotype associated with exercise and heat shock, it is likely that the predominate role in the progression of vascular disease is protective under these circumstances.

Both heat shock and exercise increase the vascular content or alter the phosphorylation status of various Hsp and both of these conditions are associated with anti-inflammatory states [14, 132]. Although exact mechanistic activities are often difficult to identify, activation of HSF1, which is the primary transcription factor involved in Hsp induction, may directly reduce general inflammation in vascular tissue [133], but most effects are probably through HSF1-induced increases in expression of Hsp [134] (see Figure 2(b)).

Hsp25/27 and Hsp70 can directly stimulate anti-inflammatory cytokines [135, 136], while Hsp70 can inhibit release of a variety of inflammatory cytokines including TNF*α*, HMGB1, and IL6 and IL1*β* [137–139]. This Hsp modulation of cytokine profile also reduces the presence of cell adhesion molecules and thereby leucocyte infiltration of the vascular wall [140, 141]. Hsp may reduce oxidative stress by a variety of mechanisms including facilitation of antioxidant pathways [10, 142, 143]. Of course the reduction in oxidative stress helps maintain NO bioavailability and reduces peroxynitrite formation [120]. In addition, increased Hsp90 in the vasculature has a direct positive effect on eNOS activation [115, 144–146], thereby maintaining vascular function (see Figures 2(b) and 2(c)).

## 9. Regulatory Role of Hsp on Apoptosis

As the inflammatory process progresses, a progressive cycle of intima expansion occurs with the death, proliferation, and migration of smooth muscle cells [147]. Accumulating lines of evidence suggest that Hsp intervene at multiple locations to inhibit cell death pathways including inhibition of death receptor signaling. Hsp25/27, 70, and 90 are involved in suppression of the mitochondria-dependent apoptosis, by directly limiting cytochrome c release [148, 149] and activation of various caspases [150, 151], by inhibiting caspase-independent pathways [152], and by inhibition of stress [153] and cell death receptor pathways [154, 155]. Hsp directly impacts on this pathway in several ways (Figures 2(b) and 2(c)). Hsp70 and Hsp25/27 can directly interact with IKK*α*, stabilizing it and preventing the inflammatory activation of the NF-*κ*B pathway [156–158]. These effects appear to be dose and time dependent [159]. Secondly, the stabilization of the cytoskeleton and antiproliferative effects of Hsp25/27 processes negatively influenced by LDL [11] may inhibit inflammation-induced vascular damage [160]. Lastly, although the effects of Hsp on vascular health have been separated by the individual Hsp involved, there is evidence that effective vascular protection requires interaction of multiple types of Hsp [161].

## 10. Metabolic Disorders and the Stress Response

Metabolic disorders, including hyperglycemia, hypercholesterolemia, hypertriglyceridemia, modified low density lipoproteins (LDL), and insulin resistance, are often associated with diabetes, metabolic syndrome, vascular inflammation, and atherosclerotic cardiovascular disease. Hyperglycemia interrupts the colocalization of Hsp90 and eNOS in endothelial cells, which may affect the production of NO and endothelium-dependent vascular relaxation [162]. Circulating levels of Hsp60 are correlated with triglycerides and small dense LDL in patients with untreated periodontitis [4]. Restriction stress increases the production of Hsp70, MCP-1, PAI-1, and monocyte adhesion but decreases adiponectin in mice [163]. The levels of Hsp27 antigen and antibody in serum of diabetic patients are associated with cardiovascular complications and insulin resistance [164]. Oxidized LDL (oxLDL) has been considered as a circulating marker for coronary artery disease [165]. Glycation increases lipid peroxidation of LDL [166]. The levels of glycated LDL (glyLDL) and oxLDL are increased in diabetic patients [167]. GlyLDL treatment increases the abundance of HSF1 and Hsp70 in endothelial cells [128]. GlyLDL or oxLDL increases the binding of HSF1 to the PAI-1 promoter and PAI-1 expression in endothelial cells [128, 168]. PAI-1 is not only a physiological inhibitor of tissue and urokinase plasminogen activator but also a marker for inflammation. Reduced fibrinolytic activity is associated with coronary artery disease and diabetic vascular complications [169]. Elevated levels of PAI-1 were detected in acute and chronic inflammatory conditions [170, 171]. Increased levels of circulatory PAI-1 have been considered as a marker of inflammation. However, the precise role of PAI-1 in inflammation remains to be determined. Antioxidants inhibit oxLDL or glyLDL-induced increases of HSF1, PAI-1, and ROS in endothelial cells, which suggests that oxidative stress may play a regulatory role in metabolic stress-induced activation of the stress response and vascular inflammation [168]. In certain stress conditions, such as massive bleeding and wounds, HSF1-mediated PAI-1 production may be protective for the body through its prothrombotic and antifibrinolytic effects. However, chronic elevation of PAI-1 production induced by metabolic disorders may lead to thrombotic tendency and ischemic events. GlyLDL or oxLDL impairs activities of mitochondrial respiratory chain enzymes in vascular endothelial cells [172, 173]. OxLDL induced oxidative stress, activation of HSF1 [168], and apoptosis and the imbalance between caspase-3 and Bcl-2 in endothelial cells [174]. The role of HSF1/Hsp in metabolic disorders-induced vascular inflammation and injury remains to be further investigated but as noted above appears to be both pro- and anti-inflammatory.

## 11. Inflammatory Imbalance in Type 1 Diabetes and Effects of Exercise

The major underlying mechanism for insulin deficiency in type 1 diabetes is *β*-cell destruction induced by an autoimmune response. Imbalance between autoreactive Th1 lymphocytes and protective Th2 lymphocytes is found in type 1 diabetes, which leads to both proinflammatory cytokines (IL-2, IL-12, TNF-*β*, and IFN-*γ*) and anti-inflammatory cytokines (IL-4, IL-6, IL-10, and IL-13) [175]. Interactions between proinflammatory cytokines (TNF-*β* and IFN-*γ*) and the receptors on membrane of *β*-cells may activate the caspase cascade and result in apoptosis. TNF-*β* and IFN-*γ* may also activate macrophages, which leads to the release of TNF-*α*, IL-1*β*, NO, and superoxide, which may increase oxidative stress and downregulation of Bcl-2, which activate NF-*κ*B and *β*-cell apoptosis leading to insulin deficiency [176]. Active macrophages may increase iNOS activity. Elevated NO generation in *β*-cells may cause oxidative stress, insulin resistance, and *β*-cell damage [177]. Oxidative stress may reduce insulin secretion from *β*-cells through stimulating the expression of uncoupling protein 2 (UCP2). UCP2 may inhibit electron transport in mitochondria and increase ROS production. Prolonged hyperglycemia may increase UCP2 in *β*-cells, which may contribute to insulin deficiency in both type 1 and type 2 diabetes [178]. Relatively less literature is available on the impact of exercise on the clinical outcome or inflammatory mediators in type 1 diabetic animals or humans. A recent study demonstrated resistance exercise before aerobic exercise improved glycemic stability throughout exercise and reduced postexercise hypoglycemia in type 1 diabetic patients [179]. Exercise induced less increase of Hsp70 in insulin-deficient diabetic rats than in control rats [180]. The impact of exercise on inflammatory mediators and the relationship with glucose metabolism in type 1 diabetes remain to be more fully investigated.

## 12. Conclusion

Both exercise and metabolic stress activate HSF1/Hsp pathway in the body. Transient stress responses induced by regular and moderate exercise tend to downregulate vascular inflammation and protect vessels from injury. Chronic stress responses induced by metabolic disorders upregulate inflammatory mediators, which leads to vascular inflammation, apoptosis, and injury. The HSF1/Hsp-mediated stress response to exercise and metabolic disorders play, distinguishable and possibly opposite roles in vascular inflammation, which may be related to the involvement of different types of Hsp, body temperature, or shear stress of blood flow. The consequences of stress responses induced by exercise and metabolic disorders, particularly of autoimmune diseases such as type 1 diabetes, on vascular inflammation require further investigation.

## Figures and Tables

**Table 1:** Heat shock protein nomenclature. Comparison of the old molecular-weight-based names with the new nomenclature as outlined in Kampinga et al. [18].

Weight-based nomenclature	New name
Hsp20	HSPB6
Hsp22	HSPB8
Hsp25/27	HSPB1
*α*A-crystallin	HSPB4
*α*B-crystallin	HSPB5
Hsc70 (cognate isoform)	HSPA8
Hsp70, Hsp72 (inducible isoform)	HSPA1A
Hsp90	HSPC1
Hsp110	HSPH2
